# OSAHS Growth Impairment and Resolution after Adenotonsillectomy in Children

**DOI:** 10.22038/IJORL.2022.57642.2986

**Published:** 2022-05

**Authors:** Antonina Mistretta, Domenico Michele Modica, Alessandro Pitruzzella, Stefano Burgio, Francesco Lorusso, Sebastiano Billone, Carla Valenti, Giulia Vita, Salvatore Poma, Marta Amata, Pietro Vita, Salvatore Gallina

**Affiliations:** 1 *Otolaryngology Unit, Department of Biomedicine and Advanced Diagnostic, University of Palermo, Palermo, Italy.*; 2 *Otorhinolaryngology Unit, Villa Sofia-Cervello Hospital, Palermo, Italy.*; 3 *Consorzio Universitario Caltanissetta, Caltanissetta, Italy.*; 4 *Department of Biomedicine Neuroscience and Advanced Diagnostic, University of Palermo, Palermo, Italy.*; 5 *Clinica Pediatrica Università degli Studi di Palermo, Palermo, Italy.*; 6 *Department of Biomedicine and Internal and Specialistic Medicine (DIBIMIS), University of Palermo, Palermo, Italy.*

**Keywords:** Adeno-tonsillectomy, Adenotonsillar hypertrophy, GH. IGF-1, Growth failure, Obstructive sleep apnoea syndrome

## Abstract

**Introduction:**

One of the most important complications of OSAHS in children is growth delay. The aim of this study was to investigate changes in clinical body growth, and laboratory growth in children with OSAHS after adeno-tonsillar surgery.

**Materials and Methods:**

In our study, among 102 children suffering from sleep-disordered breathing, 70 met the inclusion criteria because they were affected by OSAHS and adenotonsillar hypertrophy. In total, 96 children affected by adeno-tonsillar hypertrophy (55 males and 41 females) underwent nocturnal cardiorespiratory monitoring with Embletta MPR, monitoring for post-operative 24 hours. Patients underwent blood sampling to evaluate preoperative GH and IGF-1 serum levels, “placement” in Cacciari’s growth charts and adenotonsillectomy and saturation monitoring for post-operative 24 hours. According to auxological parameters, 82.86% of the patients were below the fiftieth percentile of BMI Cacciari’s growth charts and IGF-1 preoperative serum levels were below the normal range. All patients underwent adenotonsillectomy.

**Results:**

All 70 patients recovered from OSAHS according to the results of nocturnal cardiorespiratory monitoring after six months. IGF-1 serum levels significantly increased after three months and one year after. All the auxological parameters showed a significant increase after surgery. We calculated the average annual growth in height of the patients before and after adenotonsillectomy (AT): the growth rate was impaired by OSAHS (5.4±1.3 cm/year), while in the following year post-surgery we found a significant growth speed acceleration (9.9±1.7 cm/year, P=0.001).

**Conclusions:**

In conclusion, growth delay in children can be caused by OSAHS, and when it is due to adenotonsillar hypertrophy, adenotonsillectomy is to be considered as the therapy of choice.

## Introduction

Obstructive Sleep Apnoea Hypopnea Syndrome in children is defined as a “disorder of breathing during sleep characterized by prolonged partial upper airway obstruction (hypopnoea) and/or intermittent complete obstruction (obstructive apnoea) that disrupts normal ventilation during sleep and normal sleep patterns”(1). Hypopneas and apneas are associated with reduction in the normal levels of oxygen saturation in arterial blood and arousal during sleep. 

OSAHS is also associated with habitual (nightly) snoring (often with intermittent pauses, snorts, or gasps), disturbed sleep, and daytime neurobehavioral problems. Daytime sleepiness is uncommon in young children (1), unlike in adults where it is one of the most important symptoms but it can affect many organs, the most affected being the cardiovascular and nervous system (2). 

Obstructive sleep-disordered breathing (oSBD) is a common condition in the paediatric population, with an estimated prevalence of primary snoring in children ranging from 8 to 27% and of OSAHS from 1 to 5% (3,4).

Hypertrophy of the tonsils and adenoid tissue is thought to be the most common cause of oSDB in children; it causes partial or complete obstruction of the airway especially during sleep by narrowing the airway when the muscles of the pharynx relax (5). 

In adults, the pathophysiology of sleep apnoea and anatomy of the upper airways are more complex, and surgery is recommended as an option for the treatment of OSAS when non-invasive treatments such as CPAP or oral appliances have not been successful or are not tolerated (2,6,7).

One of the important consequences of OSAHS is sleep fragmentation. That is why quality of sleep and all its related functions and skills are compromised (8). One of these nocturnal functions is growth hormone (GH) secretion. GH is a peptide hormone synthesised and secreted by the pituitary gland. The sleep-onset GH pulse is caused by a surge of hypothalamic GHRH release, which coincides with a circadian-dependent period of relative somatostatin disinhibition. Extensive evidence indicates the existence of a relationship between SW sleep and increased GH secretion and, conversely, between awakenings and decreased GH release (9). GH promotes growth mostly through inducing the synthesis of insulin-like growth factor type 1 (IGF-1 or somatomedin C), a polypeptide hormone with endocrine, paracrine, and autocrine effects that shares structural homology with insulin. The great complexity of GH-IGF-1 axis is related to the wide range of growth disorders corresponding to different pathophysiological circumstances. In addition to GH deficiency (GHD) or GH excess, a multitude of other growth disorders within the GH-IGF axis have emerged until now (10). GH acts on multiple cell types, tissues and organs, but for growth, its main targets are the liver and the epiphyseal plates in long bones and spine. In the liver, GH leads to activation of Janus kinase 2 (JAK2) and signal transducer and activator of transcription 5B (STAT5B), leading to increased expression of genes encoding IGF1, IGF2, IGF-binding protein 3 (IGFBP3), IGFBP5 and acid labile subunit (ALS) which enter the circulation in the form of the so-called ternary complex. Circulating IGF1 serves as a negative factor for GH secretion in the pituitary gland and has a general anabolic function on almost all cell types. IGF1 and IGF2 (but also GH directly) stimulate the chondrocytes in the epiphyseal plate. Proliferative and hypertrophic chondrocytes in autocrine regulation also secrete IGFs (10).

GH secretion consists of constant and variable peaks: i) the constant ones take place during sleep stages 3 and 4; ii) the episodic ones coincide with the consumption of proteic meals or physical activity (11). Sleep fragmentation and the consequent poor quality of sleep stages lead to an abnormal GH-secretion (8).

The aim of this study was to investigate changes in clinical body growth (BMI, weight, height) and laboratory growth (GH and IGF-1) in children with OSAHS after adeno-tonsillar surgery. 

## Materials and Methods

The study was performed between January 2016 and May 2018, at the Otorhinolaryngology Department of the University of Palermo, evaluating 102 children (60 males; 42 females), aged between 3 and 7 years, with symptoms suggestive of oSDB. 

All these patients underwent diagnostic exams that was made up of four initial steps:

1. Clinical history: parents were asked about children’ symptoms like snoring, episodes of sleep apnoea (if noticed), daily hyperactivity, attention deficit, previous upper or lower airways pathologies, number of episodes in a year of tonsillitis and/or adenoiditis and other comorbidities.
2. Collection of auxological parameters: height (portable Harpenden stadiometers were used for height), weight (measured, when possible, in minimal underclothes to the nearest 100g on portable and properly calibrated scales) and BMI (according to the formula: weight (kg)/ height (m^2^)) -current and referable to about 12 months prior-.
3. ENT basic assessment: oral examination, including the evaluation of the morphology and possible hypertrophy of the tonsils (classified according to Brodsky score(12)), tongue, teeth, uvula and palate. Anterior rhinoscopy. Facial morphologic evaluation.
4. Fibre endoscopy: to evaluate the presence of adenoid hypertrophy (classified according to Gelardi and Cassano score(13)).

Six children were excluded from this study because they suffered from: 

• Obesity (4 children),

• Micrognathia (1 child),

• Craniofacial malformations (1 child).

In total, 96 children affected by adeno-tonsillar hypertrophy (55 males and 41 females) underwent nocturnal cardiorespiratory monitoring with Embletta MPR (Copyright © 2014 Embla Systems, Natus Medical Incorporated division) recording:

- abdominal and thoracic effort (respiratory excursion),
- nasal pressure,
- nasal flow,
- oronasal thermistry,
- snore,
- oxygen saturation and Pulse Rate,
- 1 x ExG,
- body position,
- activity,
- 1 x DC channel,

In order to assess the following parameters:

- Apnoea/hypopnoea index (AHI).
- Oxygen desaturation index (ODI).
- Lowest SpO2.
- Baseline SpO2.
- Snoring.
- Body position.
- Number of desaturations <90%.

The presence of OSAHS (Mild: AHI ≥ 1≤ 5, moderate: AHI ≥ 5.1 ≤ 10, severe: AHI ≥10) at cardio-respiratory monitoring was detected in 70 children (42 males and 28 females, mean age 4.95 ± 1.15 years). These children, affected by OSAHS and suffering from adeno-tonsillar hypertrophy (clinical examination showed adenotonsillar hypertrophy in 100% of patients, with a pharyngeal tonsils hypertrophy grade III (25.72%) and IV (74.28%) and also adenoids hypertrophy grade III (18.58%) and IV (81.42%)) met all the inclusion criteria of the study.

They underwent:

1. Blood sampling to evaluate preoperative GH and IGF-1 serum levels.

2. “Placement” in Cacciari’s growth charts (14).

3. Adenotonsillectomy (AT) and saturation monitoring for post-operative 24 hours. GH and IGF-1 dosages were made using IMMULITE® 2000 XPi system, through an enzyme immunoassay technique.

Three months later, children underwent:

1. ENT examination.

2. Blood sampling to evaluate postoperative GH and IGF-1 serum levels.

Six months after surgery, children underwent:

1. ENT examination.

2. Nocturnal cardiorespiratory monitoring with Embletta MPR.

Twelve months after surgery, children underwent:

1. ENT examination.

2. Collection of auxological parameters.

3. “Placement” in Cacciari’s growth charts. (14).

4. Blood sampling to evaluate GH and IGF-1 serum levels one year after surgery.

Parents gave their informed consent for the surgical procedure and for all the performed tests. The local Ethics Committee approved the study protocol.

Auxological parameters have been evaluated through standard deviation scores (SDS) (Z scores). Statistical analysis has been assessed applying both logistic regression and Pearson’s correlation. 

Statistical analysis was performed using SOFA Statistics (v 1.5.3) software. The software has been lunched in Windows 10 Pro (64bit), version 1909. All the above-mentioned analysis have been considered statistically significant for P < 0.05. 

## Results

Among the 70 children who met the inclusion criteria of this study, according to the results of the nocturnal cardio-respiratory monitoring, we found:

• 14 (20%) affected by mild OSAHS.

• 33 (47.2%) affected by moderate OSAHS.

• 23 (32.8%) affected by severe OSAHS.

PREOPERATIVE RESULTS

The mean values of the most important parameters assessed during preoperative evaluation were: 

• AHI mean value = 9.6/h.

• ODI mean value = 8.4/h.

• Low sat O2 mean value = 87%. 

Mean values of preoperative examination of GH and IGF-1 serum levels were:

- IGF-1: 131.4 ± 11.8 ng/ml.
- GH: normal age range.

Preoperative evaluation of auxological parameters showed:

- Weight mean value in kg: 18.5 ± 4.6.
- Height mean value in cm: 110.4 ± 9.93.
- BMI mean value in kg/m2: 15.08 ± 2.31.

Preoperative height, weight and BMI values in OSAHS children were lower compared to the mean percentile values of the same-age children in Cacciari’s charts. Only 12 children of 70 (17.14%) were above the fiftieth percentile of BMI growth charts, preoperatively. 

POST-OPERATIVE RESULTS

**Fig 1:**
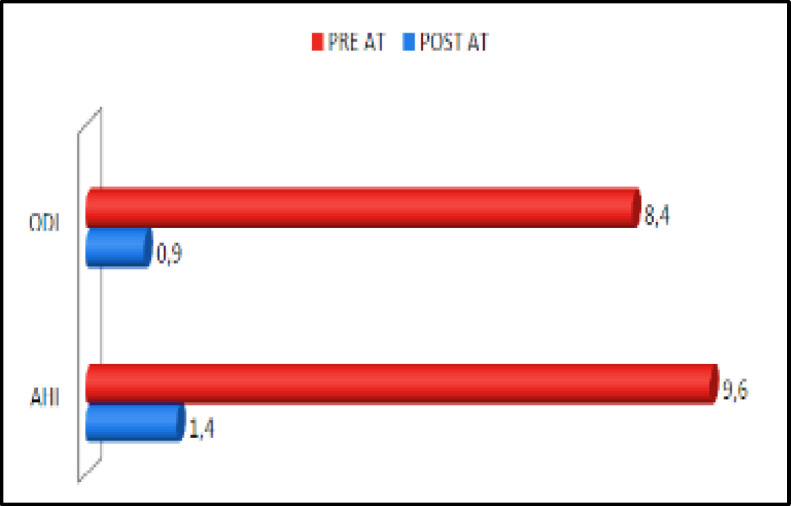
Comparison chart between preoperative and postoperative AHI and ODI mean values

After 6 months from surgery, the mean values of the most important parameters (ODI and AHI) of the nocturnal cardio-respiratory monitoring were compared: all 70 patients recovered from OSAHS, as it’s shown in the following histogram (Fig. 1).

Mean values of postoperative examination of GH and IGF-1 serum levels after 3 and after 12 months were:

- IGF-1, 3 months after: 252.3 ± 51.2 ng/ml (p-value 0.001).

- IGF-1, 12 months after: 301.4 ± 49.8 ng/ml (p-value 0.003).

**Fig 2:**
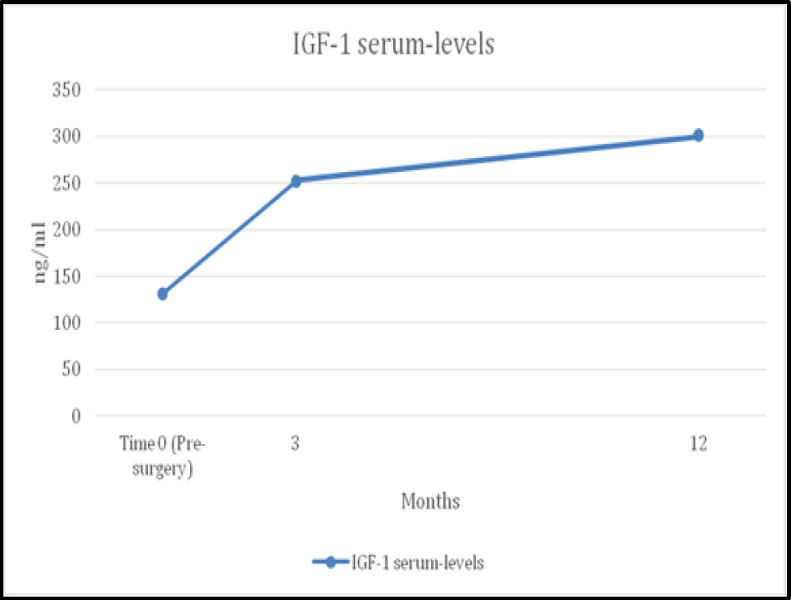
IGF-1 serum levels mean values evolution from time 0 (pre-surgery) to 12 months after surgery

There is a peak in the values ​​after three months and a subsequent slower but steady ascent phase of the values in the next nine months. (Fig.2).

Post-operative evaluation of auxological parameters (12 months after surgery) showed:

- Weight mean value in kg: 25.0 ± 5.00 (p-value <0,001).
- Height mean value in cm: 121.1 ± 9.53 (p-value <0,001).
- BMI mean value in kg/m2: 16.97 ± 2.48 (p-value <0,001).

Below, table 1 represents a comparison between preoperative and one-year post-operative parameters mean values and their SDS (Table 1).

Postoperative height, weight, and BMI values in OSAHS children were between the fiftieth and the ninetieth percentile compared to mean percentile values of same-age children in Cacciari’s charts. 

GH values were normal before surgery and did not increase significantly after adeno-tonsillectomy (p-value 0.06).

Finally, thanks to the collaboration of the family paediatricians, we collected anthropometric data of 12 months before the surgery, so we were able to calculate the average annual growth of our sample before and after adenotonsillectomy (Table 2).

**Table 1:** Comparison between preoperative and post-operative (one year follow up) mean values of auxological parameters

**Parameters**	**Mean values**	**SDS (Z-scores)**
Preoperative height (cm) Postoperative height (cm)-follow-up	111.9 ± 9.51 121.1 ± 9.53 P=<0.001	-0.26±0.9 4.3E^-16^±1 P=<0.001
Preoperative weight (kg) Postoperative weight (kg)-follow up	19.9 ± 4,4 25.0 ± 5,02 P=<0.001	-1,5E^-16 ^± 1 7.95 ± 1 P=<0.001
Preoperative BMI (kg/m^2^) Postoperative BMI (kg/m^2^)-follow up	15,78 ± 2,38 16,97 ± 2,48 P=<0.001	-1.1E^-15^ ± 1 7.9E^-16^ ± 1 P=<0.001

**Table 2:** Average annual growth of children before and after adenotonsillectomy

**Auxological parameters**	**Preoperative average annual growth values**	**Postoperative average annual growth values**
Height increase (cm/1 year)	5.4±1.3	9,9±1.7 P=0,001
Weight increase (kg/ 1 year)	2.8±2.1	5,9±1.9 P=0,003
BMI increase (kg/m^2^/ 1 year)	0,8±1.1	1,8±2.1 P=0,003

**Fig 3:**
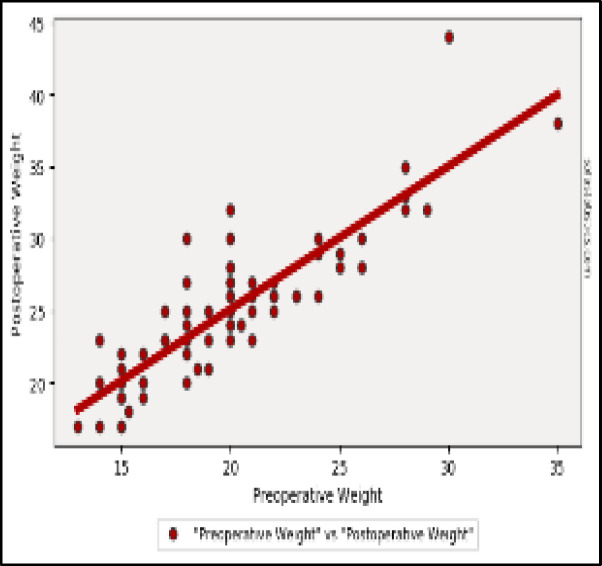
Weight: Two-tailed p value: < 0.001 (1.678e-22) 1. Pearson's R statistic: 0.869. Degrees of Freedom (df): 68. Linear Regression Details: 2. Slope: 0.992.Intercept: 5.275

**Fig 4:**
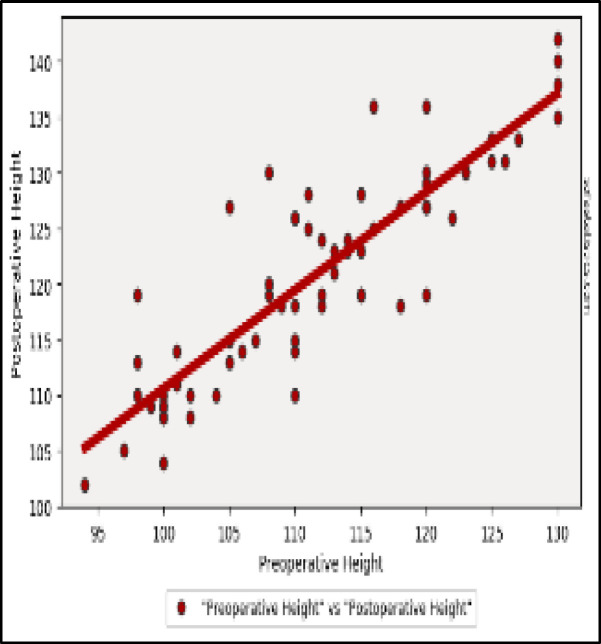
Height: Two-tailed p value: < 0.001 (5.925e-24) 1.Pearson's R statistic: 0.882. Degrees of Freedom (df): 68. Linear Regression Details: 2. Slope: 0.884. Intercept: 22.202

**Fig 5:**
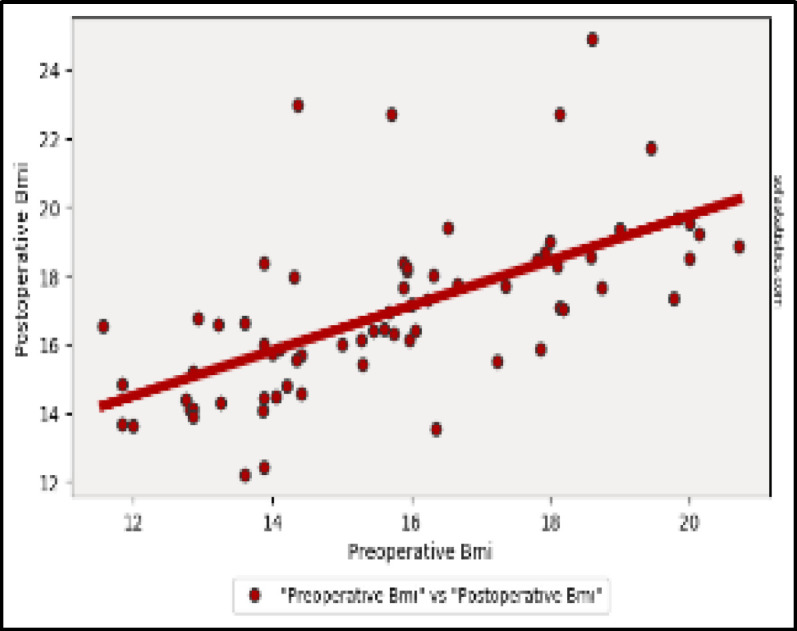
BMI: Two-tailed p value: < 0.001 (5.292e-9) 1. Pearson's R statistic: 0.63. Degrees of Freedom (df): 68. Linear Regression Details: 2. Slope: 0.657. Intercept: 6.607

All the auxological parameters show a significant increase after surgery (Fig.3, 4 and 5). 

## Discussion

OSAHS compromises children’s quality of life (15,16), from different points of view. 

Tonsillectomy plus adenoidectomy have been widely recognised as the most effective cure for OSAHS and adenotonsillar hypertrophy-affected children who suffered from it and (17,18). Many techniques have been developed recently to make this surgery quick and safe (19). In “CHAT” study, surgical treatment for OSAHS in school-age children, compared with a strategy of watchful waiting, did reduce symptoms and polysomnographic findings, thus providing evidence of beneficial effects of early adenotonsillectomy (20).

Many studies have demonstrated the various complications of OSAHS in children. Untreated paediatric OSAHS impairs neurocognitive and behavioural functions and attention ability in children, with a significant improvement after AT (21-26). Retarded growth is a well-known complication in children affected by OSAHS; AT during the prepubertal age has been shown to improve growth and its main parameters (27-29). Adenotonsillar hypertrophy and oSDB may be classified as unrecognized risk factors in the aetiology of growth failure, thus daily monitoring from otolaryngologists can play an important role in identifying and curing growth failure in children (30). In our study, we accurately selected non-obese children affected by OSAHS, but 58 children among the 70 who met all the inclusion criteria (82.86%, p-value 0.001) were below the fiftieth percentile of BMI growth charts preoperatively. This value confirms that OSAHS can cause retarded physical growth in children. The exact aetiology of growth failure remains unknown, although many different causes have been proposed: i) poor appetite and difficulties in swallowing; ii) nocturnal hypoxemia and respiratory acidosis; iii) increased caloric expenditure caused by increased work of breathing during sleep and iv) sleep fragmentation and impairment of GH-IGF1 axis (8,9,29-31). Moreover, Nieminen et al. concluded that children with OSAHS had a significant increase in BMI compared with patients who underwent AT for primary snoring, underlying that OSAHS was the most probable cause of growth failure in these children (28).

Weight gain after AT, according to Smith et al.(32), is variable depending on age. The data they collected suggest that young children, many of whom were normal weight or underweight prior to the AT, had a significant weight gain after the procedure, while those who were overweight before the AT, remained, on average, overweight after the procedure. A randomized controlled trial of 2014 (33) showed a significant weight increase in children who suffered from OSAHS, 7 months after AT. The sample of this study included overweight children at baseline. That’s why the authors could underline that AT normalizes weight in children who had failure to thrive or impaired growth, but it can also increase risk for obesity in overweight children. Thus, they suggest that the risk for worsening overweight and obesity after AT should be included into the preoperative evaluation for children at high risk(34). In the present paper, obesity was an exclusion criterion and the children included were underweight (82.86%) and normal weight (17.14%); one year after surgery, weight mean values and respective SDS z-score were significantly increased (P=<0.001).

A study of 2015 (35) included seventeen cases of non-obese children with OSAHS with a special control group: their identical twin sibling, who had no signs of OSAHS and who had higher height and weight values. Including identical twins in this study let the authors exclude the normal growth bias of other papers (genetic, family, environmental factors, for example). They confirmed that after AT not only weight but also height increased, although they underlined that their values were still lower than the control group (while the results of PSG indicated no sleep problems after surgical intervention). In our paper, height, weight and BMI values of the four subgroups got over the fiftieth percentile one year after surgery. Tahara et al.(36) showed in their study that height SDS was significant between the preoperative period and postoperative 24 months and continued over the entire 24-month follow-up period. A study of Stradling et al.(37) showed a potential growth speed acceleration reporting growth rates of 9.7 cm/year in treated children at 6 months after surgery and 7.5 cm/year in the control group. We calculated the average annual growth of our sample before and after AT: the growth rate was impaired by OSAHS (5.4±1.3 cm/year) with a significant increase after surgery showing growth speed acceleration during the year following AT (9.9±1.7 cm/year, P=0.001). In contrast, Bar et al.^ (29)^ and Williams et al.(27) stated a non-significant increase in height SDS/percentile at post-operative period. According to Tahara et al.(36), those findings might be related to the differences of national standard growth curve of each countries or to the different age in which AT was performed. They divided their sample by age in “poor improved” group (6.0±1.5 years) and in “good improved” group (4.7±1.3 years) because they found out that height velocity acceleration after AT (in a 24-months follow-up) ended earlier in the first group and they confirmed that height gain after AT, is variable depending on age.

IGF-1 is considered the main mediator of the growth-promoting actions of GH (10). Several studies showed that IGF-1 levels were found to increase significantly and reach the values of the healthy controls.(28,29,35,38) In this paper, GH levels were normal in children before and after surgery. IGF-1 serum levels showed significant increase after AT, with a particular “spike” gain after three months (131.4 to 252.3 ng/ml p-value 0.001), and a stable but less elevated increase in the following nine months (301.4 ng/ml p-value 0.003). This trend is also confirmed in other studies (35-38). 

## Conclusions

OSAHS affects children's health in many ways. Growth failure has been widely recognised as one of the consequences of OSAHS in children, with impairment of whole auxological parameters: height, weight, BMI and IGF-1 serum levels. We analysed non-obese, non-syndromic children, in whom adenotonsillar hypertrophy and upper airways narrowing was the main problem, and where adenotonsillectomy was the best treatment to perform. After AT, all the auxological parameters and their SDS values showed a significant increase in children after one-year-follow-up. These results confirm that OSAHS can cause low IGF-1 serum levels and consequent growth impairment in children. When OSAHS is caused by adenotonsillar hypertrophy, AT has to be considered as the most effective therapy in non-obese, non-syndromic children. Otherwise, in overweight children, AT can increase the risk of obesity, suggesting that after surgery parents and doctors should monitor children weight, encouraging a balanced diet and physical activity. Moreover, we suggest considering OSAHS as a possible cause of growth failure in children that do not exhibit other specific endocrinological or genetic pathologies.
